# Protocol for a randomised controlled feasibility study examining the efficacy of brief cognitive therapy for the Treatment of Anxiety Disorders in Adolescents (TAD-A)

**DOI:** 10.1186/s13063-019-3295-6

**Published:** 2019-04-25

**Authors:** Lucy Taylor, Polly Waite, Brynjar Halldorsson, Ray Percy, Mara Violato, Cathy Creswell

**Affiliations:** 1 School of Psychology and Clinical Language Sciences, University of Reading, Harry Pitt Building, Earley Gate, Reading, RG6 6AL UK; 2 Nuffield Department of Population Health, University of Oxford, Richard Doll Building, Old Road Campus, Oxford, OX3 7LF UK; 3 Departments of Experimental Psychology and Psychiatry, Anna, Watts Building, Oxford, OX2 6GG UK

**Keywords:** Brief, Adolescent, Cognitive, Treatment, Anxiety disorders, Young people, Psychological therapy, CBT

## Abstract

**Background:**

Anxiety disorders affect a quarter of the population during their lifetime, and typically emerge in childhood or adolescence. Anxiety disorders disrupt young people’s social, emotional and academic development and in the absence of treatment, often follow a chronic course. Although effective treatments, such as Cognitive Behaviour Therapy (CBT), exist, only a small proportion of adolescents with anxiety disorders who need treatment receive them. Barriers to treatment provision include the fact that CBT typically requires 14–16 sessions by a highly qualified therapist and services are stretched – resulting in lengthy waiting lists and limited access to treatment. This highlights the importance of developing new ways of providing effective treatments for adolescent anxiety disorders. This study aims to assess the feasibility of a future, large-scale trial. This will give a clear indication of the likely success of running a randomised controlled trial to compare a new, brief cognitive therapy treatment to an existing CBT group therapy for adolescents with anxiety disorders.

**Methods/design:**

The study will examine whether a definitive trial can be conducted on the basis of a feasibility RCT using a number of well-defined criteria. The feasibility RCT is a single-centre, randomised control trial. Forty-eight Young people (age 11–17.5 years) attending a university research clinic, who meet the diagnostic criteria for a DSM-5 anxiety disorder, will be randomly allocated to receive either (1) Adolescent Cognitive Therapy for Anxiety (ACTA), which involves six 60–90-min sessions and a booster session or (2) group CBT, which involves eight 2-h sessions and a booster session. As part of the feasibility indicators, patient outcomes, expectations and experiences, as well as health economic factors, will be assessed before, at the end of treatment and at a 3-month follow-up.

**Discussion:**

The successful delivery of a future, definitive trial has the potential to bring direct benefits to young people and their families, adolescent mental health service providers, as well as benefits to adult mental health services and society more broadly by disrupting the negative trajectory commonly associated with adolescent anxiety disorders.

**Trial registration:**

ISRCTN, ID: ISRCTN86123204. Retrospectively registered on 23 November 2017.

**Electronic supplementary material:**

The online version of this article (10.1186/s13063-019-3295-6) contains supplementary material, which is available to authorized users.

## Background

Anxiety disorders affect a quarter of the population during their lifetime and the majority will first be affected in childhood or adolescence, with a median onset age of 11 years [1]. Anxiety disorders are among the most frequently occurring mental health difficulties in childhood and adolescence [2]. If left untreated, they are associated with significant lifelong costs in terms of increased risks of subsequent anxiety, depression, illicit drug dependence, educational underachievement and reduced earnings [3, 4]. In addition, for some anxiety disorders, onset before the age of 20 years, compared to an older onset, is associated with greater severity and worse course [5]. This highlights the importance of effective and accessible interventions for adolescents with anxiety disorders.

Currently, the most commonly delivered treatment approach for adolescents with anxiety disorders is Cognitive Behaviour Therapy (CBT) [6], typically involving between 10 and 16 weekly treatment sessions with a specialist clinician delivered either individually (e.g. [7]) or in groups (e.g. [8–10]). However, fewer than one in five adolescents in need of treatment receives appropriate psychological interventions [11], with many facing significant delays or spending months on waiting lists for treatment within routine clinical services [12].

In order to improve access to effective psychological interventions, briefer versions of CBT have been developed that can be delivered by non-specialists, so that more intensive treatments can be reserved for those who do not, or who are unlikely to, benefit from a brief treatment [13]. Suitable brief CBT treatments have been developed and evaluated for pre-adolescent children [14]; however, there has been limited research attention on brief CBT interventions for adolescents with anxiety disorders. As far as we are aware, there are no established psychological treatments for adolescents with anxiety disorders that are less than eight sessions (Baker H, Waite P, Karalus J, Creswell C: A meta-analysis of psychological treatments for adolescents with anxiety disorders, in preparation).

Meta-analyses have demonstrated that around 60% of children and adolescents are free of their primary diagnosis at the end of CBT [6]. However, there is some (albeit mixed) evidence that adolescents with anxiety disorders have significantly lower remission rates, compared to anxious pre-adolescent children (e.g. [15]). This may be related to adolescents having more severe anxiety, higher levels of primary social anxiety disorder and comorbid depression than children [16], which are all poor prognostic indicators in treatment [15, 17].

The proportion of adolescents in remission at the end of CBT is considerably lower than those typically seen in adults who have received disorder-specific cognitive therapy for an anxiety disorder, where remission rates range from 71 to 86% post treatment and 71–85% 12–15 months after treatment [18–20], even using brief versions of the treatment [21]. Disorder-specific cognitive therapy focusses on the maintenance mechanisms that relate to specific anxiety disorders (e.g. self-focussed attention for social anxiety disorder and intolerance of uncertainty for generalised anxiety disorder). Treatment involves the development of an individualised disorder-specific cognitive model and testing out beliefs through behavioural experiments. To date, only one study has examined the applicability and effectiveness of disorder-specific individual cognitive therapy adapted for use with adolescents with anxiety disorders – showing promising results [22]. The aim of the current study is to extend previous research by developing a *brief*, individual, cognitive therapy treatment (Adolescent Cognitive Therapy for Anxiety – ACTA). This follows the principle of cognitive therapy as outlined above but also involves some adaptations; for example, involvement of family members and school as needed. Prior to adoption by Child and Adolescent Mental Health Services (CAMHS), it is essential to establish, through a randomised control trial (RCT), whether this approach brings clinical and/or economic benefits compared to the current standard form of CBT (delivered through a group) that is typically provided to adolescents with anxiety disorders.

### Current trial

In order to maximise the likelihood of a successful, large-scale RCT with a novel treatment, it is essential to explore the retention and dropout rates and the acceptability of both proposed treatment arms, and ensure that the outcomes identified are appropriate; this will be done by conducting the proposed feasibility study. Differences in outcomes between the two arms will not be analysed in any detail at this stage. The proposed study will evaluate the feasibility of a substantive RCT to compare ACTA to generic group-CBT treatment for adolescents with anxiety disorders. As anxiety disorders present a risk for ongoing mental health problems, impaired educational performance, restricted employment and productivity, and increased medical needs, the successful delivery of a future, definitive trial has the potential to bring direct benefits to young people and their families, adolescent mental health service providers, as well as adult mental health services and society more broadly by disrupting this negative trajectory.

## Methods/design

### Aims and objectives

The study aims to determine the feasibility of an RCT to assess the use of brief cognitive therapy compared to an existing group-CBT treatment for adolescents with anxiety disorders. The study will examine whether a definitive trial can be conducted on the basis of a feasibility RCT which aims to:

- Identify appropriate clinical outcome and economic measures for a subsequent definitive trial
- Explore the acceptability of the treatments and trial procedures
- Establish likely recruitment rates
- Establish the likely rate of treatment dropout
- Establish likely retention to research assessments post treatment and at 3-month follow-up
- Establish if ACTA can be delivered so that it is clearly distinct from an existing treatment, with high levels of fidelity by practitioners and credibility with patients in both arms
- Conduct exploratory analyses of possible outcomes for the two treatments including changes in anxiety symptoms, diagnostic status, quality of life, healthcare resource use and other outcomes identified through Patient and Public Involvement (PPI), and
- Describe negative impacts of the treatments and the trial procedures (to patients, their parent/s and clinicians)
- Assess young people’s outcomes on measures of symptom and functional impairment

### Feasibility criteria

The outputs from the proposed research will provide a clear indication of the feasibility of a future, definitive trial and, if indicated, the critical resources that will be required and key information to inform the design and maximise the successful completion of the trial. In order to feel confident that a definitive trial can be delivered we would require the following criteria to be met (1) serious negative impacts (e.g. worsening of symptoms, significant increase in risk as determined by clinical judgement of the treating clinician) do not occur as a result of participation in the trial; (2) there are no serious concerns about the acceptability of the trial procedures; (3) a generalisable sample can be recruited which will maintain study equipoise (i.e. at least 80% of eligible participants will agree to randomisation); (4) treatment dropout rates will be no more than 20%; (5) at least 80% of participants will complete all assessments, including a longer-term follow-up (to maximise generalisability for a larger trial); and (6) treatment delivered within the ACTA and group-CBT treatment arms will be clearly distinct in a manner that indicates therapist adherence to the manuals (with sessions containing at least 80% ‘allowable’ and less than 20% ‘not-allowable’ features of the prescribed treatment).

### Design

This study is a single-centre, parallel-design RCT comparing ACTA to eight sessions of group CBT, taken from an established intervention (‘Cool Kids “Chilled” Adolescent Anxiety Programme’) [23] in treating adolescents with anxiety disorders within the Anxiety and Depression in Young people (AnDY) Research Clinic. Appendix 1 shows the schedule of self-report measures to be completed pre-treatment, on a sessional basis, mid-treatment, post treatment and at 3-month follow-up. Treatment integrity will be assessed on the basis of video-recordings of treatment post treatment. Young people and their parents’ expectations of treatment will be assessed prior to treatment initiation using a brief questionnaire [24]. Additionally, qualitative interviews will be conducted with a subsample of participating young people and parents post treatment and thematic analysis (for young people’s interviews) and Interpretative Phenomenological Analysis (IPA) (for parent interviews) will be used to explore their experience of treatment and the research process.

### Setting

A total of 48 participants (24 in each arm) will be recruited following referral from primary and secondary care services for an assessment and treatment at the Anxiety and Depression in Young People (AnDY) Research Clinic at the University of Reading, a clinical service that receives referrals from primary and secondary care services and is funded by local NHS commissioning. The AnDY Research Clinic offers assessments, treatment and research to children and young people who are experiencing difficulties with anxiety and/or depression.

### Participants

Inclusion and exclusion criteria for the trial are as follows:

#### Inclusion criteria

Young people (aged 11–17.5 years at intake) whose primary presenting disorder is a *Diagnostic and Statistical Manual of Mental Disorders, Version 5* (DSM-5) [25] diagnosis of Separation Anxiety Disorder, Specific Phobia, Social Anxiety Disorder, Panic Disorder, Agoraphobia or Generalised Anxiety Disorder. This will be assessed using structured diagnostic interviews conducted in the clinic at baseline.

#### Exclusion criteria

1. Young people with comorbid conditions that are likely to interfere with treatment delivery, such as an established autistic spectrum disorder, learning disabilities, suicidal intent or recurrent or potentially life-limiting self-harm (i.e. current frequency of at least once per week or self-harm that requires medical attention)
2. Young people whose diagnostic assessment at baseline identifies a current primary disorder other than an anxiety disorder (such as major depressive disorder (MDD))
3. Young people who have been prescribed psychotropic medication, unless the dosage has been stable for at least 2 months
4. Young people identified by social services as currently ‘at risk’ due to, for example, child protection concerns
5. Young people who are currently receiving a psychological intervention

### Procedure

**Fig. 1:**
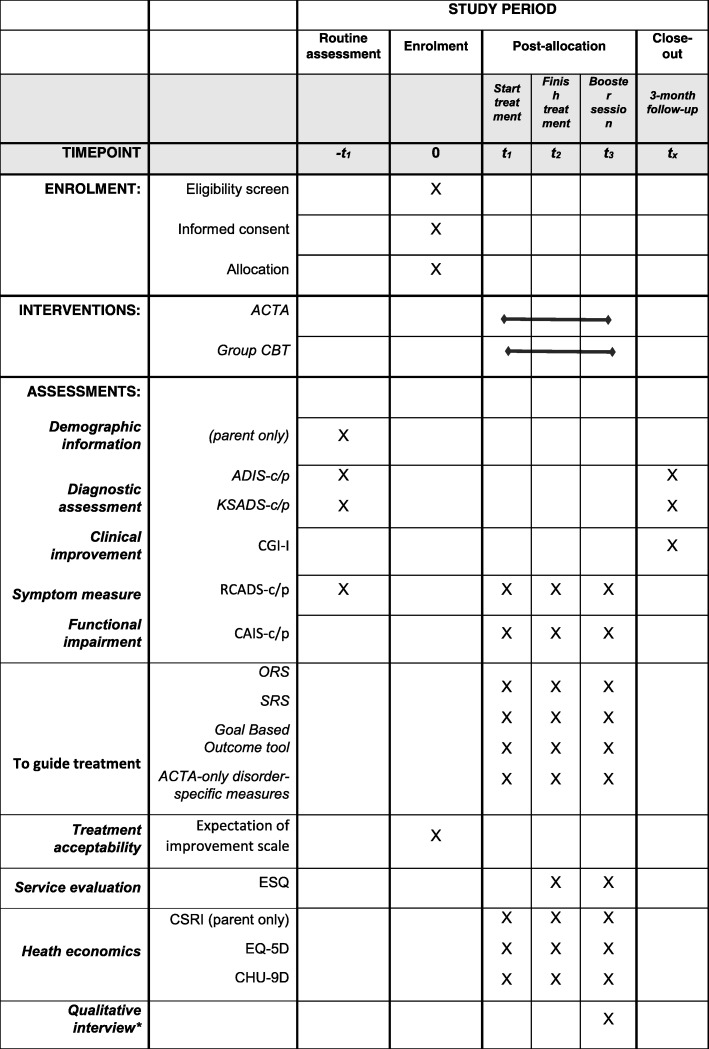
Standard Protocol Items: Recommendations for Interventional Trials (SPIRIT) schedule of enrollment, interventions and assessments. *ACTA* Adolescent Cognitive Treatment for Anxiety, *ADIS-c/p* Anxiety Disorder Interview Schedule for *Diagnostic and Statistical Manual of Mental Disorders, Version 4.* (DSM-IV) child and parent version, *KSADS-c/p* Kiddie Schedule for Affective Disorders and Schizophrenia – child and parent version, *CGI-I* Clinical Global Impression-Improvement, *RCADS-c/p* Revised Child Anxiety and Depression Scale – child and parent versions, *CAIS-c/p* Child Anxiety Impact Scale – child and parent version, *ORS* Outcome Rating Scale, *SRS* Session Rating Scale, *ACTA-only disorder-specific measures* Cognitive questionnaires (Social Anxiety Disorder = Child & Adolescent Social Cognitions Questionnaire; Generalised Anxiety Disorder = Metacognitions Questionnaire for Children; Specific Phobia (including vomit phobia) = Phobia Beliefs Questionnaire; Panic Disorder = Agoraphobia Cognitions Questionnaire), *Safety Behaviour questionnaires* (Social Anxiety Disorder = Social Behaviours Questionnaire; Generalised Anxiety Disorder = Worry Behaviour Inventory; Specific Phobia (including vomit phobia) and Panic Disorder = Safety Seeking Behaviours Questionnaire), *Symptom measures* (Social Anxiety Disorder = Liebowitz Social Anxiety Scale; Generalised Anxiety Disorder = Penn State Worry Questionnaire; Specific Phobia = Specific Phobia – Interference and Avoidance Questions, Specific Phobia of Vomiting = Specific Phobia of Vomiting Inventory; Panic Disorder = Panic Disorder Severity Scale), *ESQ* Experience of Service Questionnaire, *CSRI* Client Services Receipt Inventory, *EQ5D* EuroQol (Quality of Life), *CHU-9D* Child Health Utility (Paediatric Quality of Life), *Qualitative interviews will take place on the same day as the booster session or at another time between the finishing treatment and the 3-month follow-up

**Fig. 2:**
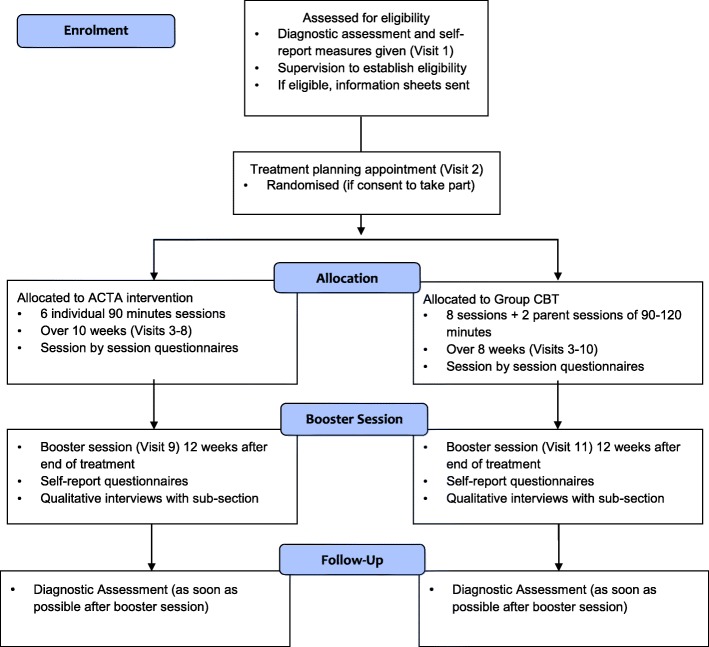
Overview of the Treatment of Anxiety Disorders in Adolescents (TAD-A) study procedure

The study procedure is in line with the Standard Protocol Items: Recommendations for Interventional Trials (SPIRIT) Statement 2013 [26] (see also Additional file 1: SPIRIT Checklist). Figure 1 displays the schedule of enrollment, interventions and assessments according to the SPIRIT Statement. Figure 2 presents an overview of the study procedures.

### Recruitment

All young people attending the clinic receive a routine clinical assessment to ascertain whether they have a primary anxiety or depressive disorder. Both young people and their parent/s or carer/s undergo a diagnostic assessment with trained assessors. Assessments involve both the young person and their parent/s being seen separately to undertake a diagnostic assessment of the adolescent. Assessments will be carried out by honorary assistant psychologists who are trained to reliability and will receive supervision for every assessment from a clinical psychologist (or equivalent) with extensive experience of delivering and supervising diagnostic assessments and proven reliability. Adolescents and their parent/s/carer/s will also be asked to independently complete self-report measures, reporting on the adolescent’s symptoms. If the young person is eligible for the trial, they will be sent information leaflets prior to a treatment planning appointment, where the results of the diagnostic assessment and treatment plan are fed back to, and discussed with, the young person and parent/s/carer/s. This appointment is always at least 24 h after providing the information leaflets. At the appointment, a member of the trial research team will discuss the study with the young person and their parent/s/carer/s, address any queries, and ensure that they understand the information provided, with particular reference to their right to withdraw throughout the study. If they agree to participate, written informed consent will then be given by the parent/s and the young person (or assent for young people under 16 years of age). Screening logs will be maintained for eligible participants not recruited, to inform acceptability of the study to young people. Reasons for non-participation in the trial will be collected anonymously.

### Randomisation

Consenting participants will be randomised to receive individual sessions of cognitive therapy (ACTA) or group-CBT sessions from the Cool Kids ‘Chilled’ Group treatment [23]. Simple randomisation will be adopted by way of numbered, sealed envelopes prepared before recruitment commences. The allocation sequence will be determined using computer-generated random numbers. In order to minimise bias, the researcher allocating the participant will be blind to the contents of the envelope. Participants will be informed of their allocation immediately following their consent to take part in the study.

### Treatment

Once randomised, participants will be allocated to a clinician for the relevant treatment arm. Clinicians delivering the trial interventions will be psychological wellbeing practitioners or clinical psychologists and will only deliver treatment in one arm of the trial. The clinician will either arrange treatment session dates with the family (in the case of ACTA), or inform the family of the dates when the next CBT group will run (in the case of Chilled). A letter will also be sent to the participant’s general practitioner (GP) to inform them of the young person’s participation in the research study. For the group, all treatment sessions will take place at the AnDY Research Clinic. For ACTA, treatment sessions will take place at the clinic, but later on in treatment, sessions may take place off-site (e.g. at school, in a café or on public transport) in order to facilitate meaningful behavioural experiments.

### Follow-up

After the 3-month booster session, participants in the trial will have a follow-up diagnostic and clinical assessment. These will be conducted by trained assessors who are blind to the treatment arm to minimise any potential bias. Clinical supervision will be provided by a skilled and competent senior assessor who is similarly blind to the treatment arm of the participant. For participants who have discontinued with the treatment they were allocated to at randomisation, this follow-up assessment will be conducted at the time when it would have occurred had they continued in that treatment arm. Some young people and/or their parent/s/carer/s will be invited to take part in a qualitative interview to discuss their experiences of receiving treatment and being part of the research study. Interviews will be conducted by postgraduate students who have had training in qualitative research and will receive supervision from researchers with expertise in this approach. A purposive sampling strategy will be adopted to identify participants for qualitative interview with the aim of including participants that differ on demographic variables and treatment outcomes (Smith, 1998).

### Intervention

#### ACTA: Adolescent Cognitive Treatment for Anxiety

This treatment has been developed at the Anxiety and Depression in Young People (AnDY) Research Clinic based on the principles of cognitive therapy. The approach taken is based on work of Beck [27, 28] and then further developed in the UK by members of a research group originally based in Oxford, including David Clark, Paul Salkovskis, Adrian Wells and colleagues (e.g. [29–34]) and for generalised anxiety disorder, by researchers in Quebec [35, 36]. Sessions include (1) the development of a disorder-specific model based on the person’s own beliefs, safety behaviours and symptoms; (2) testing beliefs through behavioural experiments involving the person experiencing feared situations while dropping their safety behaviours (so not using a habituation rationale) and (3) the development of a blueprint at the end of treatment. There are also disorder-specific interventions that were developed for the adult treatments and used within this treatment, such as video feedback for social anxiety disorder and worry-awareness training for generalised anxiety disorder. Treatment is guided by routine outcome measures. Clinicians will be trained in delivering the therapy by two senior clinicians who are experienced in delivering cognitive therapy for anxiety disorders, as well as, attending weekly group supervision sessions (each lasting 90 min) and watching videos of trained clinicians delivering the treatment. The treatment is briefer than standard cognitive therapy (typically around 12–16 sessions) and involves six sessions of between 60 and 90 min delivered over 10 weeks (on weeks 1, 2, 3, 4, 6 and 10), with a further booster session 12 weeks after the end of treatment.

#### ‘Chilled’ Group: Cool Kids ‘Chilled’ Child and Adolescent Anxiety Programme

The Adolescent version of the Cool Kids Child and Adolescent Anxiety Programme [23], known as ‘Chilled’, is a well-established anxiety management programme that teaches CBT techniques for managing anxiety. This has been adapted to be delivered in eight group sessions lasting from 90 min to 2 h (rather than ten 90-min sessions as outlined in the treatment manual), supplemented by the two parent sessions as per the treatment manual (a total of 20 h). Topics that are covered include: psychoeducation, thoughts and feelings, realistic thinking, exposure, managing emotions, problem-solving and, at the final session, relapse prevention. Treatment will be delivered by two clinicians in small groups (ideally with between four and six young people), each week over 8 weeks. The two parent sessions are delivered concurrently to the adolescent sessions on weeks 3 and 8 by one of the clinicians. There is also an additional booster session for the adolescents, 12 weeks after the end of treatment.

### Measures and assessment

In addition to the diagnostic assessments that will be conducted at screening (baseline) and after the 3-month booster session, parent/s/carer/s and adolescents will complete paper copies of questionnaires, reporting on the young person, using anonymised unique identifying numbers. Measures will be completed prior to treatment (pre-treatment), at the end of the main treatment sessions (post treatment) and following the 3-month booster session (3-month follow-up). The young person will also complete measures prior to each treatment session. The pre-treatment measures and the ones completed prior to each session are completed at home. The post-treatment and follow-up measures are completed in the clinic, or, where not able to be done, may be taken home and posted back to the clinic. A detailed schedule for when each measure is used is provided in Appendix 1.

Demographic information will be collected from the parent/s on the pre-treatment questionnaire and this will include information about the young person (age, gender, ethnicity, treatment and/or medication for psychological difficulties) and the parent/s (relationship to young person, age, relationship status, education (self and partner), employment (self and partner)). This will be used to describe the sample.

#### Diagnoses of anxiety disorders and comorbid disorders

The diagnostic assessments at baseline and 3-month follow-up will use the following interview schedules to establish if the young person reaches diagnostic criteria for anxiety and mood disorders. The Anxiety Disorders Interview Schedule (ADIS) – child and parent report (ADIS-c/p; [37]) is a structured diagnostic interview which will be administered to young people and their parent/s by highly trained research assistants (psychology graduates) trained to a high level of inter-rater reliability. All final diagnoses and Client Services Receipt (CSRs) will be determined by consensus with a supervisor with proven reliability. The Anxiety section of the ADIS-c/p assessment is used to determine whether the young person meets the diagnostic criteria for an anxiety disorder, behavioural disorder and other comorbid anxiety disorders and to establish a clinician rating of severity for each disorder (CSR). The pre-treatment diagnosis with the highest CSR will be classed as the primary diagnosis. Additionally, mood disorders will be assessed using the relevant sections of the Kiddie Schedule for Affective Disorders and Schizophrenia (K-SADS; [38]) which is a structured diagnostic interview for *Diagnostic and Statistical Manual of Mental Disorders, Version 4* (DSM-IV) affective disorders and schizophrenia.

#### Symptoms of anxiety and depression

The Revised Child Anxiety and Depression Scale (RCADS; [39]) will be used to measure symptoms of anxiety disorders and depression. This will be completed at pre, post and follow-up appointments by young people and parent/s/carer/s, and additionally by the young people at every treatment session. The RCADS is a 47-item parent and child report scale which assesses symptoms of Separation Anxiety Disorder, Social Anxiety Disorder, Generalised Anxiety Disorder, Panic Disorder, Obsessive Compulsive Disorder and Major Depressive Disorder. Responders rate how often each item applies on a scale of 0 (‘never’) to 3 (‘always’). The RCADS has been shown to have robust psychometric properties in children and young people from 7 to 18 years of age [40].

#### Functional impairment

The Child Anxiety Impact Scale (CAIS; [41]) will be used to determine the extent to which anxiety interferes in the young person’s life. This will be completed at pre, post and follow-up appointments by young people and parent/s/carer/s. This measure covers three psychosocial domains (academic, social activities and home/family environments) and consists of 27 items rated on a 4-point scale There are versions for children/adolescents and parent/s to complete, both of which have been shown to have good psychometric properties [41, 42]. Internal consistency for the CAIS-c/p was good to excellent across assessment time points (CAIS-C *α* = .85–99; CAIS-P *α* = .93–95).

The Clinical Global Impression Scale-Improvement (CGI-I; [43]) will be used after the 3-month follow-up assessment to assess the young person’s post-treatment changes in global functioning. This asks the clinician to rate how improved the patient is compared to their initial assessment, prior to treatment, on a scale of 1 (very much improved) to 7 (very much worse). Final scores will be dichotomised to represent ‘much or very much improved’ versus ‘other’. A second rater will independently rate the CGI-I for all participants in order to establish inter-rater reliability.

#### Disorder-specific measures (ACTA only)

Young people in the ACTA treatment arm will also complete up to three disorder-specific measures (for their primary anxiety disorder) to measure symptoms, cognitions and safety behaviours, in order to guide the treatment sessions. The cognitions measure for each disorder will be administered at each treatment session. Symptom and safety behaviour measures will be administered pre-treatment, mid-way through treatment, post treatment and at the booster assessment. Measures that have been designed for use with adults will be adapted for use with adolescents on the basis of consultation with young people. A detailed list of the measures used can be found in Appendix 2.

#### Session-by-session measures to guide treatment (both treatments)

The Outcome Rating Scale (ORS; [44]) will be used to assess functioning across different areas of the young person’s life. It has four items: symptom distress, interpersonal wellbeing, social role and overall wellbeing. Each item is rated using a ten-centre visual analogue scale, with instructions to place a mark on each line. A higher score indicates better functioning. It has good reliability and validity with an adolescent population [45].

The Session Rating Scale (SRS; [46, 47]) assesses key dimensions of an effective therapeutic relationship and is given at the end of each therapy session to obtain feedback from young people and parent/s/carer/s so that any issues related to therapeutic alliances can be immediately identified and addressed. It comprises four rating scales (relationship with the therapist, goals and topics, approach or method and an overall rating) and uses the same visual analogue scales as the ORS. It has well-established reliability and validity [47, 48].

The Goal Based Outcomes tool (GBO; [49]) enables the young person to set up to three goals at the beginning of treatment as a way of evaluating their progress. Progress towards individual goals is then periodically rated on a scale from 0 (no progress) to 10 (goal has been reached). Although this measure is now widely used in CAMHS, its psychometric properties have not yet been established.

#### Service satisfaction

At the end of treatment and at the 3-month follow-up assessment, participants will rate their satisfaction with the service that they have received using the Experience of Service Questionnaire (ESQ; [50]), a measure that was developed by the Health Care Commission as a means of measuring service satisfaction in CAMHS. There are versions for young people and their parent/s/carer/s to report on the extent to which they agree with 12 statements looking at what the respondent liked about the service, what they felt needed improving, and three free-text sections for any other comments. It is routinely used within CAMHS and has been demonstrated to have good psychometric properties [51].

#### Health economic measures

Health economic measures as detailed below are collected from parent/s and young people on the pre, post and 3-month follow-up self-report questionnaires. Clinicians will use logs at each treatment and supervision session and any other times as required.

A societal perspective for costs will be adopted and patient-level resource use data will be collected from parent/s/carer/s on a Client Services Receipt Inventory (CSRI) using patient-health diaries to facilitate recall of healthcare resource use and also from clinicians and supervisors on Economic Logs. This data will be provided by clinicians and parent(s)/carer(s) and will include all health and social care cost-generating resources (e.g. staff time for provision of treatment, training and supervision, GP use, referrals and other relevant services identified), non-NHS cost-generating services (e.g. educational services) as well as leisure and lost productivity time estimates for the parent/s/carer/s (e.g. days off school/college/work).

The EuroQol (Quality of Life) (EQ-5D-5 L) [52] is a well-validated preference-based measure of health-related quality of life, designed to estimate quality-adjusted life years (QALYs), that is widely used across disease areas. The EQ-5D questionnaire contains five simple questions each concerned with a different domain of everyday life, i.e. mobility, self-care, usual activities, pain/discomfort and anxiety/depression. For each domain the respondent has to indicate whether they experience no problems, slight problems, moderate problems, severe problems or extreme problems. The respondent’s answers provide a description or profile of the respondent’s quality of life, and a weight or value can then be placed on each profile using an existing UK tariff derived from the general public [52, 53]. The full questionnaire also includes a visual analogue scale (VAS) for participants to rate their overall health on a scale from 0 (‘worst imaginable health’) to 100 (‘best imaginable health’). The quality of life of carers will be assessed using the EQ-5D-5 L self-report. The EQ-5D-Y [54, 55] was adapted directly from the EQ-5D to estimate utility values for young people (from 8 years). It covers the same domains as the EQ-5D, but the wording of the questions in each dimension is modified to make it appropriate to a younger age range. Both the EQ-5D-5 L and the EQ-5D-Y have established feasibility and reliability [52, 53].

The Child Health Utility 9D (CHU-9D; [56, 57]) is a paediatric measure of health-related quality of life, which allows the calculation of QALYs for use in cost utility analysis. It includes nine dimensions (worried, sad, pain, tired, annoyed, schoolwork, sleep, daily routine, activities) each with five levels. The measure was originally developed with children aged 7–11 years, and subsequently validated in an adolescent population (11–17 years) [57, 58]. The CHU-9D is also available in a ‘proxy’ version for parent/ carer completion, and this will also be used.

#### Treatment credibility

Participant expectancies and views regarding treatment credibility will also be assessed prior to treatment through a credibility and expectancy for improvement scale [24]. This consists of three items, rated on a scale from 0 (not at all) to 10 (completely), asking about how logical the treatment seems, confidence in its success at reducing their symptoms, and their likelihood to recommend the therapy to a friend with similar symptoms.

#### Therapy Content Checklist

To establish that the therapies in each arm are distinct from one another, a checklist of the components of each therapy will be given to therapists to complete at the end of every treatment session. The checklist has been designed for this trial and has 27 items that are distinct to either ACTA (12 items, e.g. development of an idiosyncratic version of the cognitive model) or group CBT (15 items, e.g. cognitive restructuring using thought records). Therapists will indicate which components were carried out in the session that they have just completed. The ratings will be used to compare the content of the ACTA and the Chilled Group sessions in order to determine their distinctiveness.

#### Qualitative interviews

Qualitative interviews will be conducted post treatment to explore young people’s and parents’ experiences of treatment and the research process. Interviews with young people will follow a predetermined topic guide.

### Sample size

Guided by previous successful feasibility studies comparing similar interventions [59–62], the sample size of 48 (with 24 participants in each arm) is considered to be sufficient to provide an estimate of variation in outcomes (on both continuous and dichotomous variables) on which to power the definitive trial, if indicated. It is also considered sufficient to indicate if any adverse events or significant deterioration were likely to occur. As this is a feasibility study, many of the outcome measures are descriptive (e.g. recruitment rates, acceptability of treatment, dropout rates). The outcome variables will be used mainly to determine the viability of running a full-scale RCT. Any results from hypothesis testing comparing the outcome of the two treatments will be treated as preliminary and interpreted with caution as no formal power calculations have been carried out [63]. A subsample will be involved in qualitative interviews after the treatment has been delivered. We will use purposive sampling and sample according to the methodological approach; this is likely to involve around six to ten young people and around four to six parents/carers from each treatment arm.

### Data analysis

#### Analysis of clinical outcomes

Analysis of the feasibility study will primarily investigate recruitment and retention rates, presented as a Consolidated Standards of Reporting Trials (CONSORT) diagram providing both overall and individual arm results at all assessment points. Clinical outcomes will be represented using descriptive statistics for each study arm. An exploratory comparison of between-group differences will be undertaken to assess whether the observed effect size is in line with our expected effect based on the literature, using analysis of covariance or a suitable alternative. Ninety-five percent confidence intervals will be constructed for the between-group differences for each of the outcomes, adjusted for baseline, and compared with the literature. Where differences exist, further investigation of both the group means and variances will be undertaken. Data on the proportion of missing data will also be presented. Where available, the overall baseline clinical data will be compared with routinely available service-level data for adolescents with a primary diagnosis of an anxiety disorder in order to assess the representativeness of trial participants.

### Analysis of economic outcomes

Suitability and acceptability of the economic measures will be assessed on the basis of both rates of responses at the end of the feasibility study and from young people’s and their parent/s/carer/s’ feedback. Proportions of responses to healthcare resource use and health outcome measure questions will be presented in separate tables for the ACTA and the Chilled Group arms. Missing data will be explored in order to establish whether this is due to lack of response to specific questions, to the measure altogether, or to loss of follow-up. Rates of this missing data will also be compared to that of clinical measures to assess patterns in the response of certain participants. For both quality of life measures (i.e. the EQ-5D-Y and the CHU-9D), utility scores and QALYs will be calculated and compared for both treatment groups to explore how sensitive each measure is to change over time. Adolescent self-report and parent/carer report on the young person will also be compared for the CHU-9D in order to assess any discrepancies in responses of the adolescents and their parent/s. Finally, variation in quality of life as derived from the EQ-5D-5 L will be reported and compared across both treatment groups.

### Analysis of qualitative outcomes

Thematic analysis [64] will be used to identify emergent themes within the young people’s interviews. This technique was chosen due to its flexible nature, and because it is not associated with a particular theoretical framework [65]. Parent/carer interviews will be conducted by a researcher as part of a DClinPsy course and to satisfy the requirements of this course, Interpretative Phenomenological Analysis (IPA; [66]) will be used to assess this data. This analysis is phenomenological and interpretive in that it is concerned with both understanding how people make sense of their experiences and acknowledging the role of the researcher in identifying patterns of meaning across experiential accounts. Both thematic analysis and IPA have been used to explore people’s experiences of psychotherapy (e.g. [67, 68]) and are suitable for analysis of this data. A number of strategies will be employed to enhance the credibility and methodological rigour of the analysis [69], such as co-analysis of transcripts, use of reflexive practices in supervisory discussion and presentation of the analysis to a small, expert, reference group that includes adolescents and carers.

### Trial and data monitoring

As this is a feasibility study being conducted at a single, secure site, the study investigators will be responsible for monitoring the conduct of the research, including data monitoring, managing adverse events, and any decisions relating to early termination of the trial. Additionally, the trial management team, which will hold regular review meetings, will manage the safety and efficacy of the data.

## Discussion

This study has been designed to assess the feasibility and acceptability of conducting a RCT for comparing ACTA brief cognitive therapy to a group-CBT treatment for adolescents with anxiety disorders. As far as we are aware, this is the first study to examine the acceptability and applicability of a brief form of psychological treatment for adolescents with anxiety disorders. Establishing the efficacy of brief treatments is crucial to improve the number of young people being able to access appropriate psychological interventions without significant delay. It is a strength of this study that it is taking part in a clinical service that receives referrals from primary and secondary care services and is funded by local NHS commissioning and so participants are not a self-selecting population. In addition, clinicians will be predominantly psychological wellbeing practitioners, a workforce trained to deliver brief CBT treatments, and, therefore, able to provide treatment in a cost-effective manner.

If indicated, this feasibility trial will lead to a definitive RCT to establish whether this approach brings clinical and/or economic benefits compared to the current standard form of CBT that is typically provided to adolescents with anxiety disorders.

### Implications

The successful delivery of a future trial has the potential to bring direct benefits to young people and their families, adolescent mental health service providers, as well as benefits to adult mental health services and society more broadly by disrupting the negative trajectory commonly associated with adolescent anxiety disorders.

### Limitations and barriers

As this trial is a feasibility study, there will be no direct impact from this research on patient care, but it has the potential to maximise the successful completion of a future, definitive trial which will bring the benefits as detailed above. The sample size, although sufficient to determine the viability of running a full scale RCT, is not large enough to formally compare the outcomes of the two treatments. It will, however, provide an estimate of the variation on which to power a future, definitive trial. A potential barrier to a future trial is the changing landscape of mental health provision in the UK, and the ability to access suitable young people for the trial.

## Trial status

The study is currently ongoing. Recruitment of participants started in October 2017 and will continue until the target sample size is recruited. This is expected to be September 2018.

### Additional file

Additional file 1: Standard Protocol Items: Recommendations for Interventional Trials (SPIRIT) 2013 Checklist: recommended items to address in a clinical trial protocol and related documents. (DOCX 48 kb)

## Appendix 1

### Assessment schedule

#### Clinical diagnostic assessments (baseline and final assessment)

*Administered to parent(s)/carer(s) and young person at baseline screening (1) and after final 3-month follow-up questionnaire (2)*

- Administered by assessors:

ADIS-c/p (Anxiety section and common comorbid disorders)

K-SADS-c/p (Depression screen and supplement (including persistent depressive disorder), mania screen (supplement only if screening questions are endorsed))

- Self-report measure of symptoms: RCADS-c/p
- Self-report measure of functional impairment: CAIS-c/p
- Clinician rating of functional impairment: CGI-I (at follow-up assessment only)

#### Self-report questionnaire measures

For young people in the Adolescent Cognitive Treatment for Anxiety (ACTA) treatment arm, the following disorder-specific measures (according to primary diagnosis) are also given:

**Table:**

*Symptom measures – at pre, mid (after session 3), post and 3-month follow-up*	
Social Anxiety Disorder	Liebowitz Social Anxiety Scale (LSAS-c)
Generalised Anxiety Disorder	Penn State Worry Questionnaire (PSWQ-c)
Specific Phobia	Specific Phobia – Interference and Avoidance Questions
Specific Phobia of Vomiting	Specific Phobia of Vomiting Inventory (SPOVI)
Panic Disorder	Panic Disorder Severity Scale
*Safety behaviour measures – at pre, mid (after session 3), post and 3-month follow-up*	
Social Anxiety Disorder	Social Behaviours Questionnaire (SBQ)
Generalised Anxiety Disorder	Worry Behaviour Inventory (WBI)
Specific Phobia	Safety Seeking Behaviours Questionnaire
Specific Phobia of Vomiting	Safety Seeking Behaviours Questionnaire
Panic Disorder	Safety Seeking Behaviours Questionnaire
*Cognition measures – at pre, post and 3-month follow-up and at every treatment session*	
Social Anxiety Disorder	Child & Adolescent Social Cognitions Questionnaire (SCQ)
Generalised Anxiety Disorder	Metacognitions Questionnaire for Children
Specific Phobia	Phobia Beliefs Questionnaire
Specific Phobia of Vomiting	Phobia Beliefs Questionnaire
Panic Disorder	Agoraphobia Cognitions Questionnaire (ACQ)

#### Clinician measures completed during treatment delivery

Therapy Content Checklist Completed by clinicians after each treatment session

Economic Log Completed by clinicians after each treatment and supervision session and any additional contact time

## Appendix 2

### Disorder-specific measures for the Adolescent Cognitive Treatment for Anxiety (ACTA) treatment arm

#### Symptom measures

Social Anxiety Disorder – The Liebowitz Social Anxiety Scale for Children and Adolescents (LSAS-C/A; [70]) will be administered to assess adolescents’ social anxiety symptoms. The LSAS-C/A includes 24 items, rated on a scale from 0 ‘*none*’ to 3 ‘*severe*’, to assess fear and avoidance of social interaction and performance. The LSAS-C/A has well established psychometric properties when administered to children and young people from 7 to 18 years of age [71].

Generalised Anxiety Disorder (GAD) – The Penn State Worry Questionnaire for Children (PSWQ-C; [72]) will be used to assess adolescents’ GAD symptoms. The Penn State Worry Questionnaire for Children (PSWQ-C) was adapted from the adult version [73]. Participants rate 14 worry statements using a 4-point Likert scale (from 0 ‘*never true’* to 3 ‘*always true’*). It has demonstrated good psychometric properties for children between 6 and 18 years of age [72, 74].

Specific Phobia – Specific Phobia – Interference and Avoidance questions will be used to measure adolescents’ phobia symptoms and has been developed for the current study for use within the ACTA treatment. Young people rate on a 9-point scale how much their specified phobia (a) upsets or bothers them (0 *= not at all*, to 8 = *very severely disturbing/disabling*) and (b) how much this phobia causes them to avoid situations (0 = *would not avoid it*, to 8 = *always avoid it*). The psychometric properties of this measure have not been evaluated.

Specific Phobia of Vomiting – The Specific Phobia of Vomiting Inventory (SPOVI; [75]) will be used to measure symptoms associated with this specific phobia. This is a 14-item self-report measure where each statement is rated on a Likert-type scale (0 = *not at all*, to 4 = *all the time*) relating to frequency of how much that statement has affected them in the past week (e.g. ‘*I have been trying to find reasons to explain why I feel nauseous*’). The psychometric properties of this scale for use with adolescents specifically have not yet been established.

Panic Disorder – The Panic Disorder Severity Scale for Children and Adolescents [76] will be administered to assess change in the frequency and severity of adolescents’ panic disorder symptoms and anticipatory anxiety and associated agoraphobia, avoidance, fear, work and social impairments. There are seven items; each rated on a 0–4 scale, with a higher score indicated greater severity. It has been shown to have good psychometric properties with an adolescent population [76].

#### Measures of cognition

Social Anxiety Disorder – The Social Cognitions Questionnaire (SCQ; [77]) will be used to measures social cognitions and attitudes. The SCQ is a 22-item questionnaire assessing social-anxiety-related negative automatic thoughts. Each thought is rated twice. First, the respondent rates the frequency with which the thought occurred on a scale of 1 (‘thought never occurs’) to 5 (‘thought always occurs when I am anxious’). Second, the respondent rates the extent to which the thought was considered to be true on a scale of 0 (‘I do not believe this thought’) to 100 (‘I am completely convinced this thought is true’). The SCQ has high internal consistency and discriminant validity in adults [77] and has been used with adolescents [22]. The current version has been further adapted for use with children and adolescents on the basis of consultation with young people, in that wording has been amended to be more developmentally appropriate on seven items and a further seven items have been added.

Generalised Anxiety Disorder (GAD) – The Metacognitions Questionnaire for Children (MCQ-C; [78]) will be used to measure cognitions associated with GAD. It is a 24-item scale and comprises four subscales: (1) positive meta-worry, (2) negative meta-worry, (3) superstition, punishment and responsibility beliefs and (4) cognitive monitoring. Participants are asked to indicate how much they agree with each statement on a 4-point scale, from ‘do not agree’ at one extreme, and ‘agree very much’ at the other. It has good psychometric properties for use with adolescents [78].

Specific Phobia and Specific Phobia of Vomiting – The Phobia Beliefs Questionnaire (PBQ; (Centre for Anxiety Disorders and Trauma. Phobia Beliefs Questionnaire, unpublished) will be administered to measure cognitions associated with phobia disorders. This was developed at the Centre for Anxiety Disorders and Trauma (CADAT) for use with adults (Roberts A, Kerr A: Centre for Anxiety Disorders and Trauma guide to treating specific phobia, unpublished). The PBQ is a 34-item questionnaire where the person rates on a 5-point scale how often they have a particular thought about their phobia (0 = *thought never occurs,* 4 = *thought always occurs)*. Additionally, they rate on an 11-point scale whether they believe these thoughts to be true (0 = *I do not believe this at all*, 10 = *I am completely convinced by this thought*). There is no psychometric data for this measure with adults or adolescents.

Panic Disorder – The Agoraphobia Cognitions Questionnaire (ACQ; [79]), modified by Clark and colleagues [20] will be administered to measure cognitions associated with panic disorder. This measure includes 18 cognitions commonly associated with panic disorder. First, the respondent rates the frequency with which the thought occurred on a scale of 1 (‘*thought never occurs*’) to 5 (‘*thought always occurs when I am anxious*’). Second, the respondent rates the extent to which the thought was considered to be true on a scale of 0 (‘*I do not believe this thought*’) to 100 (‘*I am completely convinced this thought is true*’). The ACQ has high construct and discriminant validity in adults [79] but to date, there is no psychometric data on the use of this measure with adolescents.

#### Measures of safety seeking behaviours

Social Anxiety Disorder – The Social Behaviours Questionnaire (SBQ; [77]), adapted for children/adolescents, will be used to measure safety behaviours associated with social anxiety disorder. The SBQ is a 28-item scale assessing the use of social-phobia-related safety behaviours when respondents are anxious or in a social situation. Each behaviour is rated on a 4-point scale ranging from, 0 = ‘*Never*’, through to 1 = ‘*Sometimes*’, 2 = ‘*Other*’ and 3 = ‘*Always*’. The SBQ has good psychometrics properties in adults [77]. It has been used in the treatment of adolescents with social anxiety disorder [22]. This version has been adapted for use with children and adolescents, with the addition of four further questions and changes to wording on four other items to be more developmental appropriate on the basis of consultation with young people.

Generalised Anxiety Disorder (GAD) – The Worry Behaviours Inventory (WBI; [80]) will be used to measure worry-related safety-seeking behaviours. This was developed for use with adults. It has 10 items and the individual rates how often they have used safety behaviours common to GAD on a 5-point scale (from 0 ‘*none on the time*’ to 4 ‘*all of the time*’). This measure has good psychometric properties in adults [81] but has not yet been evaluated with adolescents.

Specific Phobia, Specific Phobia of Vomiting & Panic Disorder – The Safety Seeking Behaviours Questionnaire (SSBQ; [82]) will be administered to identify phobia- and panic-related safety-seeking behaviours. This involves 15 items; each rated on a 4-point scale from ‘*never*’ to ‘*always*’. There is no psychometric data for this measure with adults or adolescents.

## Abbreviations

ACQ: Agoraphobia Cognitions Questionnaire
ACTA: Adolescent Cognitive Treatment for Anxiety
ADIS-c/p: Anxiety Disorder Interview Schedule for DSM-IV – child and parent version (Anxiety section and common comorbid disorders)
AnDY: Anxiety and Depression in Young People
CAIS-c/p: Child Anxiety Impact Scale – child and parent version
CAMHS: Child and Adolescent Mental Health Services
CBT: Cognitive Behaviour Therapy
CGI-I: Clinical Global Impression-Improvement (1–7)
CHU-9D: Child Health Utility (Paediatric Quality of Life)
CSRI: Client Services Receipt Inventory (recall facilitated at post treatment and 3-month follow-up session using patient health diaries)
DSM-5: *Diagnostic and Statistical Manual of Mental Disorders, Version 5*
EQ-5D: EuroQol (Quality of Life)
ESQ: Experience of Service Questionnaire
K-SADS-c/p: Kiddie Schedule for Affective Disorders and Schizophrenia (Depression screen and supplement (including persistent depressive disorder), mania screen (supplement only if screening questions are endorsed)) – child and parent versions
LSAS-C: Liebowitz Social Anxiety Scale-child report
ORS: Outcome Rating Scale
PDSS: Panic Disorder Severity Scale
PPI: Patient and Public Involvement
PSWQ –c: Penn State Worry questionnaire for children
RCADS-c/p: Revised Child Anxiety and Depression Scale – child and parent versions
RCT: Randomised controlled trial
SCQ: Social Cognitions Questionnaire
SRS: Session Rating Scale
WBI: Worry Behaviour Inventory
